# Effect of High-Intensity Ultrasound and Calcium Chelation on Functional Properties of Casein Micelles

**DOI:** 10.3390/foods15122246

**Published:** 2026-06-22

**Authors:** Mufida Khalifa Eljabali, Naaman Francisco Nogueira Silva, Behdad Shokrollahi Yancheshmeh, Heidi Olander Petersen, Federico Casanova, Aberham Hailu Feyissa

**Affiliations:** 1 Research Group for Food Production Engineering, National Food Institute, Technical University of Denmark, Søltofts Plads, 2800 Kongens Lyngby, Denmark; mukmej@food.dtu.dk (M.K.E.); beshyan@food.dtu.dk (B.S.Y.); hope@food.dtu.dk (H.O.P.); 2 Center of Natural Sciences, Federal University of São Carlos (UFSCar), Buri 18290-000, SP, Brazil; naaman.nogueira@ufscar.br

**Keywords:** casein micelles, high intensity ultrasound, casein chelation, casein functionality, casein structure

## Abstract

Functional properties of caseins play a crucial role in the dairy industry, so it is important to develop methods to improve their functionality. The aim of this study is to investigate the combined effect of high-intensity ultrasound (HIUS) treatment and calcium chelation on functional properties of casein micelles. For this purpose, micellar casein concentrate (MCC) was prepared with a concentration of 3% (*w*/*w*) casein. Then, 0 and 10 mM of Disodium hydrogen phosphate was added. HIUS was performed at a frequency of 20 kHz, power intensity of 550 W/cm^2^, and an amplitude of 100% for 0, 5, 10, 15, and 20 min at 25 °C. Factorial design was employed to investigate the effect of ultrasound time (UST) and disodium phosphate (DSP) on foam capacity (FC), emulsion activity index (EAI), gelation time (GT), *G*′ at 480 min of oscillation time (*G*′_480_), slope of complex viscosity, and linear viscoelastic region (LVR). At 0 mM of DSP, increasing UST from 0 to 15 min decreased GT from 114.39 ± 3.20 to 83.52 ± 1.61 min, and it extended LVR from 40.36 ± 0.12 to 41.27 ± 0.27% of the applied strain. In addition, applying HIUS for 15 min increased the elasticity and firmness of MCC gel networks at 0 mM of DSP. *G*′_480_ was not influenced by UST, but it was reduced by DSP from 108.40 ± 3.29 to 15.78 ± 1.58 Pa. Increasing both UST and DSP significantly increased FC from 110.00 ± 13.23 to 163.33 ± 11.55% and foam stability (FS) in all treatments. FS reached its maximum (doubled) after 10 min of UST at 0 mM of DSP. However, EAI and emulsion stability index (ESI) decreased with increasing both UST and DSP. HIUS treatment combined with calcium chelation might highlight a new approach to improve foaming properties. However, regardless of calcium chelation, HIUS treatment is a promising technology to improve the gelling properties of casein micelles.

## 1. Introduction

Milk proteins play a crucial role in the food industry due to their functional properties, such as water binding, foaming, emulsifying, and gelling properties [1]. Micellar casein concentrate (MCC) is manufactured by microfiltration (MF) of skim milk, and is commercially available as a liquid, concentrate, or dried powder containing ≥8, ≥22, and ≥80% total protein, respectively [2,3]. MCC contains mainly 90% of casein on total protein, organized in a micellar form, and a minor amount of whey protein, lactose, soluble minerals, and non-protein nitrogen (NPN) [4,5]. Functional properties of casein determine its behavior during food processing [1,6], depending on pH, temperature, ionic strength, presence of reducing agents, and physical and chemical modifications [1]. MCC has significant commercial value in the food industry because of its favorable nutritional and functional characteristics. Because of its ability for water binding, foaming, and gelling purposes, it is suitable for a wide range of food applications [1,7]. Therefore, MCC is widely incorporated into dairy products, meat products, beverages, bakery products, and specialized nutrition products such as infant formulas and sports nutrition products [8,9,10]. Micellar casein-based ingredients are commonly utilized in yogurt, fermented dairy beverages, high-protein ultra-high temperature (UHT) beverages, processed cheese, and protein-fortified formulations [2,3,11,12]. Functional characteristics of casein can be effectively improved by modifying the molecular structure. One of the common methods to modify the structure of casein is manipulating its mineral equilibrium [4,13,14,15,16]. Addition of calcium chelators to milk systems is a known technique to improve the functional properties of casein [9,15,17]. Calcium ions contribute to the internal stability of casein micelles as they form linkages between protein molecules, either as colloidal calcium phosphate (CCP) or directly bound to caseins [18,19]. Calcium chelators can alter the distribution of calcium ions between soluble and colloidal phases, which can affect the structural integrity and functionality of casein micelles [13,20,21]. Excessive addition of calcium chelators leads to a decrease in thermal stability, highlighting the need to produce modified casein [15,16]. The food industry has shown interest in the application of high-intensity ultrasound (HIUS), as an emerging technology, to improve protein functionalities [6,10,22]. In liquid media, such as milk, ultrasound diffusion generates acoustic cavitation, characterized by the formation, growth, and violent collapse of bubbles, which produces localized extreme temperatures and pressure, generating intense physical effects, including microstreaming, shear forces, turbulence, shock waves, and microjets [23,24,25,26]. HIUS, which uses frequency levels in the range of 20–100 kHz and power density in the range of 10–1000 Wcm^−2^, can be utilized in dairy applications. Several studies have investigated the functional properties of casein affected by HIUS [8,27,28,29,30,31]; however, there is a lack of deep insight into the combined effect of calcium chelators and HIUS on the properties of MCC. Therefore, the purpose of this study is to investigate the influence of HIUS treatment combined with the addition of disodium phosphate (DSP), a food-grade calcium chelator used in the dairy industry, on the functional properties of MCC. This combination might highlight a new approach to improve MCC functionality and extend its applications in the food industry.

## 2. Materials and Methods

### 2.1. Materials and Chemicals

Micellar casein powder (MCP) (Promilk 852B Via Lacta, 5% total moisture, 81.5% total protein, 85% protein on dry matter, 92% casein, 8% total ash, 5% total moisture, 4% lactose, 2.4% calcium, and 1.5% fat) was kindly provided by Ingredia (Arras, France). MCP was obtained from raw milk after skimming, followed by an initial microfiltration step using a 1.4 µm membrane to remove residual fat and microorganisms. The resulting permeate was then concentrated by microfiltration using a 0.1 µm membrane, producing a micellar casein concentrate. Subsequently, the concentrate was subjected to diafiltration with water to remove most soluble components, followed by additional microfiltration steps. Once the desired concentration was achieved, the final concentrate was directly spray-dried without any additional heat treatment [2,3,32,33]. MCP was stored in vacuumed food-grade plastic bags at 4 °C for experimental use [34,35]. Disodium hydrogen phosphate and sodium hydroxide (Sigma-Aldrich Chemie GmbH, Steinheim, Germany). (Sigma Aldrich, Steinheim, Germany), sodium azide (Sigma-Aldrich, Shanghai, China), hydrochloric acid (Fisher Chemicals, Schwerte, Germany), Glucono-*δ*-lactone (Sigma Aldrich, Milan, Italy), sodium dodecyl sulphate (Sigma-Aldrich Chemie GmbH, Steinheim, Germany), and sunflower oil were used.

### 2.2. Preparation of Samples

Micellar casein concentrate (MCC) was prepared using deionized water to obtain a suspension with a concentration of 3% *w*/*w* casein. Sodium azide was added at 0.02% (w/w) to prevent microbial growth. The suspension was stirred at 500 rpm (IKA-WERKE, GMBH and CO.KG, Staufen, Germany) at room temperature for 72 h [7,27,35]. Disodium hydrogen phosphate (DSP) was added to the suspension to obtain a final concentration of 0 and 10 mM [15,16,36]. The suspension was stirred (IKA-WERKE, GMBH and CO.KG, Staufen, Germany) for 1 h at room temperature to ensure complete dissolution. Then, the pH was adjusted to 7.0 ± 0.01 by adding 1 M sodium hydroxide or 1 M hydrochloric acid [13].

### 2.3. High-Intensity Ultrasound (HIUS) Treatment

A hundred ml from each sample was ultrasonicated in a double-jacketed glass vessel, and the temperature was kept below 25 °C. HIUS treatment was performed using a sonifier (SFX550, Branson, CT, USA), operating at a constant frequency of 20 kHz and power intensity of 550 W/cm^2^. The samples were ultrasonicated at an amplitude of 100% for 0, 5, 10, 15, and 20 min in pulsed mode (5 s ON and 5 s OFF) [7,9,26,27,35]. The energy delivered to the samples was 0, 750, 1500, 2250, and 3000 J/mL, which was calculated using Equation (1):

(1) $$AED=\frac{P\times t}{V}$$

where *AED* is acoustic energy density (J/mL); *P* represents the output power (W); *t* is total ultrasonication time (s); and *V* is the volume of the sample (mL) [7,9,10,16,37,38].

### 2.4. Foaming Properties

Twenty ml of MCC was placed in a 50 mL Falcon tube and homogenized using an Ultra turrax homogenizer (IKA-T25 digital ULTRA TURRAX, EAC, IKA-WERKE, GMBH and CO.KG, Staufen, Germany) at 10,000 rpm for 2 min at 25 °C to obtain foam. The total volume of solutions was recorded at time intervals of 0, 10, 20, 30, 40, 50, and 60 min after homogenization. Foaming capacity (*FC*) and foaming stability (*FS*) were calculated using Equation (2) and Equation (3), respectively:

(2) $$FC\;\left(\%\right)=\frac{V_{0}-V_{s}}{V_{s}}\times 100$$

(3) $$FS\;\left(\%\right)=\frac{V_{t}-V_{s}}{V_{0}-V_{s}}\times 100$$

where *V_s_* is the initial volume of the sample (20 mL), *V*_0_ is the volume of the sample immediately taken after homogenization, *V_t_* is the volume of the sample after 10, 20, 30, 40, 50, and 60 min of homogenization [6,27,28].

### 2.5. Emulsifying Properties

Emulsion activity index (*EAI*) and emulsion stability index (*ESI*) were measured by the absorbance at 500 nm as described by Zhang et al. (2018) with minor modification [8]. Five mL of sunflower oil and 20 mL of treated MCC samples (at the ratio of 1:4) were mixed. The mixture was stirred with an Ultra turrax homogenizer (IKA-T25 digital ULTRA TURRAX, EAC, Germany) at a speed of 10,000 rpm for 2 min at room temperature. An aliquot of the emulsion (50 µL) was pipetted immediately at time intervals 0, 10, 20, and 30 min and then mixed with 5 mL of 0.1% (*w*/*v*) sodium dodecyl sulphate (SDS) solution. The absorbance of the diluted emulsion was measured at 500 nm against a blank (without emulsion) using a spectrophotometer (UV-160A, UV-VISIBLE RECORDING SPECTROPHOTOMETER, MD825-24, Shimadzu, Kyoto, Japan). *EAI* was determined by the absorbance immediately measured after emulsion formation (0 min), and *ESI* was estimated by measuring the absorbance after 10, 20, and 30 min of emulsion forming [8,9,28]. Then, *EAI* and *ESI* were calculated using Equation (4) and Equation (5), respectively:

(4) $$EAI\;\left(\frac{m^{2}}{g}\right)=\frac{2\times 2.303\times A_{0}}{C\times \theta \times 10000}\times DF$$

(5) $$ESI\;\left(\%\right)=\frac{A_{t}}{A_{0}}\times 100$$

where *DF* is the dilution factor (100), *A*_0_ is the absorbance at 0 min, *A_t_* is the absorbance after 10, 20, and 30 min of emulsion formation, *C* is the protein concentration (g/mL), *θ* is the volume fraction of the oil (0.20) [28,39].

### 2.6. Gelling Properties

Oscillatory rheological measurements were performed using a rheometer (DISCOVERY HR-2, hybrid rheometer, TA Instruments-Waters LLC, New Castle, DE, USA) and geometry (vane concentric cylinder, Peltier Steel—1104115) with cup diameter of 30.4 mm, bob diameter of 28 mm, bob length 42 mm, operation gab of 4000 μm, and loading gab of 90,000 μm. The temperature was regulated by a Peltier unit, attaching a water circulation system, and kept constant at 25 °C [29,30].

#### 2.6.1. Gelling (Acid-Induced Gelation)

A predetermined amount of Glucono-*δ*-lactone (GDL) (0.1 g of GDL/g of casein) was added to 40 mL of the treated samples at room temperature with mild stirring for 1 min to ensure complete GDL solubilization. This was followed by transferring the sample into the cub of the rheometer. The samples were covered with a solvent trap to prevent evaporation [29].

#### 2.6.2. Oscillation Time

Oscillation time was determined by conducting a pre-experiment, in which pH for all treatments was tracked and recorded in intervals of 30 min until gels were formed (Supplementary Table S3). Depending on the pre-experiment results, oscillation time was performed for 480 min (8 h) at a constant frequency of 1 Hz and strain of 1%, and at 25 °C [29,30]. The first point of measurement was recorded 1 min after the start [40]. The delay is due to the equilibration step performed before starting the measurements. Changes in elastic (*G′*) and viscus (*G*″) moduli were recorded every 6 s during gel formation [30] to follow the changes in relative strength of gels [29]. Gelation time (GT) was defined as the time at which *G*′ and *G*″ cross over and tan *δ* (the ratio of *G*″ to *G′*) = 1 [29]. *G*′ value, which was recorded at the last minute of oscillation time (480 min), is defined as *G*′_480_ [41,42,43].

#### 2.6.3. Oscillation Frequency

After 480 min, an oscillation frequency test was performed on the freshly formed gel. A strain amplitude of 1% was used for the frequency sweep test to ensure that the test was performed in the linear viscoelastic region (LVR). Frequency dependence was determined by investigating elastic (*G*′) and viscus (*G*″) moduli and complex viscosity (η*) over a frequency (f) range varying from 0.1 to 10 Hz [an angular frequency (*ω*) range from 0.63 to 62.83 rad/s [29,30]. To evaluate the viscoelastic properties of the treated MCC gels, the slopes of log-log plots of η* against *ω* were calculated [30].

#### 2.6.4. Oscillation Amplitude

Following the oscillation frequency test, deformation properties of the formed MCC gels were measured by progressively increasing the strain from 0.1 to 500% until the gels were broken. *G*′ and *G*″ were monitored over strain progressing [40,41]. The region where *G*′ of the formed gels shows non-significant deviation from a constant value is defined as the linear viscoelastic region (LVR). LVR was used as a structural parameter to evaluate the firmness of MCC gels [40]. The applied strain at the end of LVR was determined [41].

### 2.7. Experimental Design and Statistical Analysis

Factorial Design was employed to investigate (screen) the effect of ultrasound time (UST) and disodium phosphate (DSP) concentration on the response variables: foam capacity (*FC*), emulsion activity index (*EAI*), gelation time (GT), *G*′ at the last minute of oscillation time (*G′*_480_), slope of log complex viscosity (*n* − 1), and the applied strain at the end of linear viscoelastic region (*γ*). The factors were varied: UST at five levels (0, 5, 10, 15, and 20 min) and DSP at two levels (0 and 10 mM). The effect of UST and DSP on response variables was described by Equation (6):

(6) $$Y=\beta_{0}+\beta_{1}X_{1}+\beta_{2}X_{2}+\beta_{1,2}X_{1}X_{2}+e$$

where *Y* represents the response variables (*FC*, *EAI*, GT, *G*′_480_, *n* − 1, and *γ*). *X*_1_ and *X*_2_ are the levels of the independent variables (UST and DSP), respectively; $\beta_{0},\;\beta_{1},\;\beta_{2},\;\beta_{1,2}$ are regression coefficients for intercept, the linear effect, and interaction terms, respectively.

Foam stability (*FS*), emulsion stability index (*ESI*), and complex viscosity (η*) were fitted to linear, exponential, and power-low models (Equations (7)–(9)), respectively:

(7) $$FS=F_{0}-bt$$

(8) $$ESI=E_{0}\;e^{\left(-ct\right)}$$

(9) $$log\;\eta *=logk-\left(n-1\right)\;log\omega$$

where *F*_0_ is the initial state of foam (%), *b* is the slope of linear fit *FS* (1/min), *t* is the time (min), *E*_0_ is the initial state of emulsion (%), *c* is the decay rate of exponential fit *ESI* (1/min), *log k* is the intercept, (*n* − 1) is the slope of η* (Pa·s), and *ω* (rad/s) is angular frequency. *b* and *c* were used to evaluate the stability of the formed foams and emulsions, respectively, while (*n* − 1) was used to evaluate the viscoelastic properties of the formed gels.

The analysis of variance (ANOVA) for the response variables was used to find the significant terms in the model. The validity of the models was evaluated by lack of fit, multiple correlation coefficient (R^2^), and adjusted R^2^ (adj-R^2^) (see Supplementary Table S1). The data was evaluated utilizing JMP Pro15 software (SAS Institute Inc., Cary, NC, USA). Results were expressed as the average and standard deviation of three independent experiments (see Supplementary Table S2).

## 3. Results and Discussion

### 3.1. Foam Capacity and Stability

**Figure 1:**
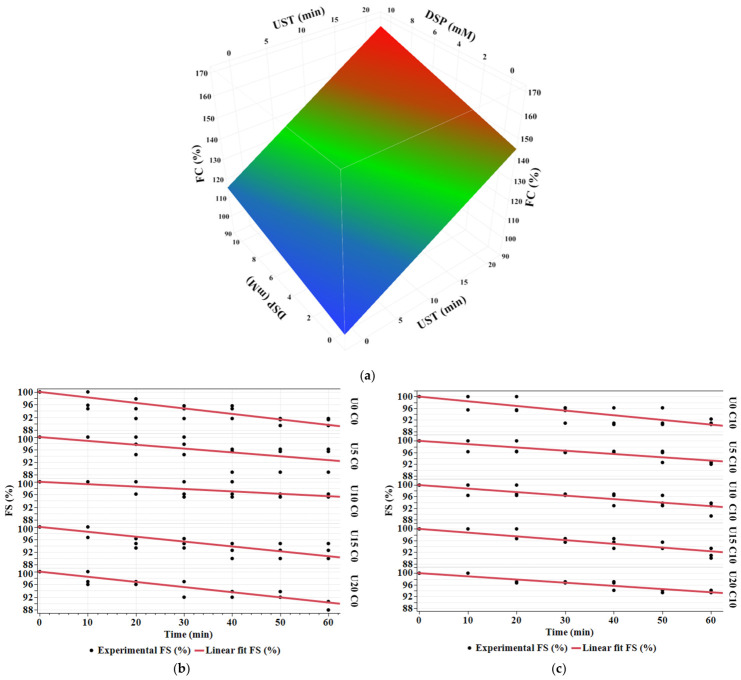
Effect of ultrasound time (UST) and disodium phosphate (DSP) on: (**a**) foam capacity (*FC*), (**b**,**c**) foam stability (*FS*) at 0 and 10 mM, respectively, [experimental (dotted) and model fit (solid line)] of micellar casein concentrate: UST (U0 = 0 min, U5 = 5 min, U10 = 10 min, U15 = 15 min, and U20 = 20 min) and DSP concentration (C0 = 0 mM and C10 = 10 mM).

Foaming properties of protein are related to its ability to adsorb on the air-water surface, at which the interfacial tension decreases [4]. Evaluation of protein foaming characteristics includes two aspects: foaming capacity and foaming stability [28]. Figure 1a demonstrates the effect of ultrasound time (UST) and disodium phosphate (DSP) concentration on foam capacity (*FC*) of micellar casein concentrate (MCC). *FC* significantly increases with increasing both UST (*p* = 0.0001) and DSP (*p* = 0.02). However, there is no significant interaction effect of UST and DSP (Supplementary Table S1). The effect of UST is more intense than the effect of DSP. *FC* rose from 110 ± 13.23 to 163.33 ± 11.55 (%) after 20 min of UST at 10 mM of DSP (Supplementary Table S2). In another study conducted by Kommineni et al. (2022), they reported that the foam capacity of 5% MCC significantly increased from 102.67 to 126.50% after 30% of calcium reduction [4]. Foaming characteristics are influenced by factors such as temperature, pH, ionic strength, concentration, and conformational structure of protein [4]. The addition of calcium chelating agents, by binding calcium ions, results in dissociation of micellar casein into the aqueous phase [20,21]. The available non-micellar casein (particularly, β-casein) would be adsorbed onto the foam surface and decrease the interfacial tension, thus improving the foamability of MCC [4]. Improved foaming capacity of milk proteins by HIUS treatment can be attributed to the mechanical effects of acoustic cavitation that induce changes in the conformation of protein structure, such as particle size and surface hydrophobicity [1]. Foam capacity was positively correlated with the results of the smaller particle size, lower surface hydrophobicity, higher solubility, and the changes in the secondary structure recorded in our previous study [44].

Foam stability (*FS*) of the formed foams was evaluated by the slope of the model fit *FS* over the measuring time of 60 min (Figure 1b,c, and Table 1). As presented in Table 1, the slopes of *FS* of all treated MCC are less than that of untreated MCC (U0 C0 treatment). The treatment U10 C0 (10 min of UST and 0 mM of DSP concentration) recorded the lowest slope of *FS* 0.075 ± 0.009 (1/min) (Table 1). This indicates that all treated MCC have higher *FS* compared to untreated MCC. Ultrasound causes unfolding of protein molecules (structural changes in the secondary and tertiary structures) [13,34] and decreases the particle size of protein molecules, which leads to an increase in their ability to adsorb on the air–water interface to encapsulate air and reduce interfacial tension, forming a continuous and stable interface film. Therefore, this results in more stable foam [1,28].

The relative changes of *FS* slopes of treated to untreated MCC were used to evaluate the *FS* of all treatments (Table 1). The relative changes in all treated MCC are less than 1, which indicates that *FS* increases by increasing both UST and DSP by over 60 min. The lowest relative change was 0.436, which was recorded by the treatment U10 C0 (10 min of UST and 0 mM of DSP concentration) (Table 1). Independent of DSP, the *FS* of MCC was doubled after 10 min of UST, after which *FS* decreased gradually with increasing UST. It has been reported that controlled ultrasound treatment of milk proteins can improve their foaming properties. However, overprocessing with ultrasonication can cause a considerable loss in their foaming properties due to protein aggregate formation induced by excessive ultrasound treatment [1].

Our findings regarding *FC* agreed with Zhao et al. (2022)’s results [27]. They studied the combined effect of ultrasound and pH-shifting on the foaming properties of 30 mg/mL of MCC treated by 20 kHz and 300 W of HIUS at 30 °C, pH 8.0, 9.0, 10.0, 11.0, 12.0, and native pH (7.17 ± 0.02) for 5 min. They reported that *FC* of MCC was significantly higher after pH-shifting combined with ultrasonication. *FC* increased from about 50% at native pH to 100% at pH 11. In contrast with our results, they found that all treated MCC showed a decrease in *FS*. pH-shifting from native pH to pH 11 led to the exposure of hydrophobic groups and then resulted in higher surface hydrophobicity of casein micelles and less *FS*. However, the present study was conducted at the neutral pH (7.0 ± 0.01), which is close to the native pH (7.17 ± 0.02) in the Zhao et al. study, which reported more *FS* compared to the other values of pH [27].

### 3.2. Emulsion Activity and Stability Indexes

**Figure 2:**
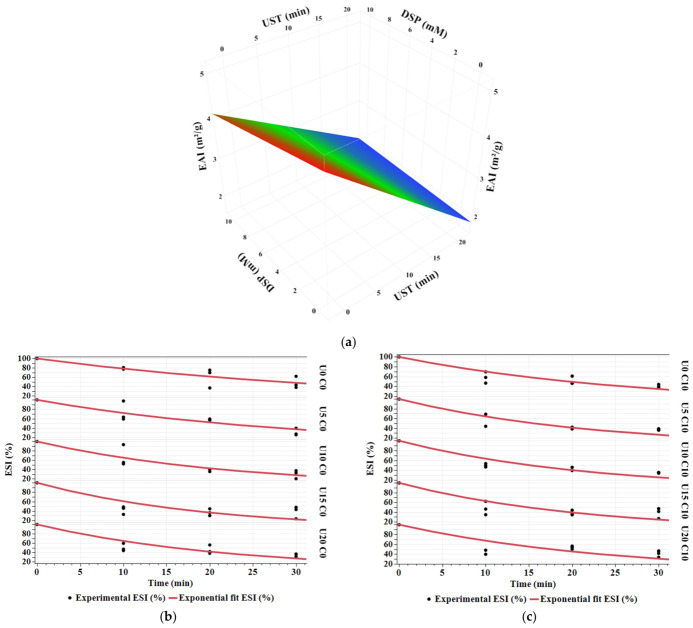
Effect of ultrasound time (UST) and disodium phosphate (DSP) on: (**a**) emulsion activity index (*EAI*), (**b**,**c**) emulsion stability index (*ESI*) at 0 and 10 mM, respectively, [experimental (dotted) and model fit (solid line)] of micellar casein concentrate: UST (U0 = 0 min, U5 = 5 min, U10 = 10 min, U15 = 15 min, and U20 = 20 min) and DSP concentration (C0 = 0 mM and C10 = 10 mM).

Emulsifying properties of protein are evaluated by emulsifying activity index (*EAI*) and emulsion stability index (*ESI*), characterizing the ability of the protein to be adsorbed on the interface of fat globules and the aqueous phase and their capacity to remain at the interface during processing and storage, respectively [1]. The effect of ultrasound time (UST) and disodium phosphate (DSP) concentration on emulsion activity index (*EAI*) of micellar casein concentrate (MCC) is represented in Figure 2a. *EAI* significantly decreases with increasing UST (*p* = 0.0003), while it is not affected by increasing DSP (*p* = 0.39) (Supplementary Table S1). After 20 min of ultrasonication, *EAI* reduced from 4.48 ± 0.34 to 2.31 ± 0.09 (m^2^/g) (Supplementary Table S2).

The slopes of the model fit *ESI* over the measuring time of 30 min and their relative changes to untreated MCC were employed to evaluate the stability of formed emulsions (Figure 2b,c, and Table 1). As presented in Table 1, *ESI* slopes of all treated MCC are higher than those of untreated MCC (U0 C0 treatment). As a result, all treated MCC recorded relative changes of *ESI* higher than 1. This indicates that *ESI* decreases with increasing both UST and DSP. Emulsifying properties are relevant to the changes in the structural properties, particle size, solubility, surface hydrophobicity, oil–water ratio, and pH [1,28,45]. Due to the high molecular structure of proteins, they diffuse slowly to the oil–water interface compared to low molecular weight emulsifiers such as tween 80 [46]. Once proteins undergo structural changes, they rearrange themselves to position their hydrophobic amino acids within the oil phase and their hydrophilic amino acids within the aqueous phase. This reduces the interfacial tension and the overall free energy, which is critical for stabilizing emulsion systems [46]. In contrast, long hydrophilic or hydrophobic fragments could adversely influence protein interfacial activity [9]. Therefore, the decrease in both *EAI* and *ESI* recorded in the present study can be interpreted by the positive correlation with surface hydrophobicity. The decrease in surface hydrophobicity with increasing UST and DSP concentration observed in our previous study [44] could reduce the ability of protein to be adsorbed and stabilized on the interface of oil droplets and water.

Zhang et al. (2018) measured *EAI* and *ESI* of 5% MCC treated by HIUS at 20 kHz and power intensity of 58 W/L for a maximum of 5 min [8]. They reported that *EAI* and *ESI* increased significantly in the first 2 min of ultrasound time. As the treatment time prolonged, no significant difference was observed for *EAI* and *ESI*. They attributed their results to the increase in surface hydrophobicity and solubility recorded after 5 min of ultrasonication [8]. In another study, Song et al. (2021) evaluated *EAI* and *ESI* of 7.52% MCC ultrasonicated at 20 kHz, an amplitude of 50%, and a power output of 453 W, at 20 and 50 °C for a maximum of 60 min [9]. They observed that *EAI* and *ESI* did not show significant changes at 20 °C controlled treatment with increasing ultrasound time from 0 to 15 min. However, increasing ultrasound time to 30 min led to significant improvement in both *EAI* and *ESI*, at which the maximum *EAI* and *ESI* were 42.3 ± 1.9 and 87.1 ± 5.1%, respectively. Their results are positively correlated with the increase in surface hydrophobicity after 30 min of ultrasonication [9]. In contrast with the present results, Kommineni et al. (2022) reported that the emulsion capacity of 5% MCC significantly increased from 63 to 66% [4]. However, emulsion stability was reduced from 87 to 81% after 30% of calcium reduction. They referred the difference in emulsion properties to the surface activity and particle size of treated MCC. The higher surface activity and/or smaller particle size, the greater emulsion properties [4].

### 3.3. Gelling Properties

#### 3.3.1. Gelation Time (GT) and Gel Strength

**Figure 3:**
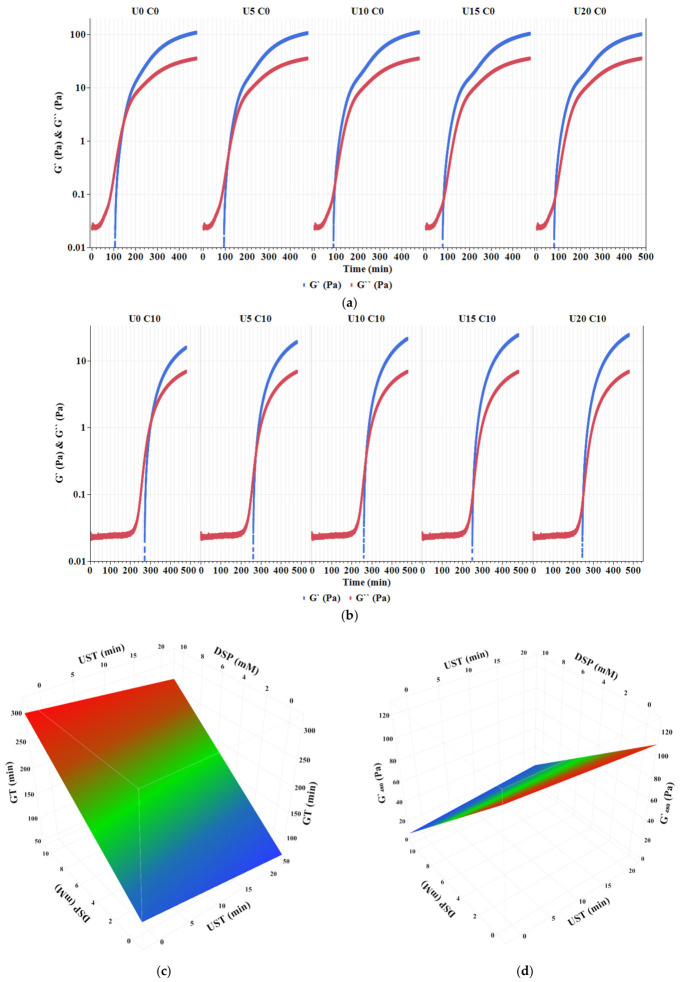
Effect of ultrasound time (UST) and disodium phosphate (DSP) on: (**a**,**b**) oscillation time profiles of elastic (*G*′) and viscus (*G*″) moduli at 0 and 10 mM, respectively; (**c**) gelation time (GT); (**d**) *G*′ at the end of oscillation time (*G*′_480_) of micellar casein concentrate: UST (U0 = 0 min, U5 = 5 min, U10 = 10 min, U15 = 15 min, and U20 = 20 min) and DSP concentration (C0 = 0 mM and C10 = 10 mM).

Acid-induced gel can be formed by the gradual decomposition of glucono-*δ*-lacton (GDL) into gluconic acid [43]. In the pre-experiment, pH values of all treatments were recorded in intervals of 30 min during acidification time (480 min). Gels were formed at pH values ranging from 4.87 ± 0.1 to 4.98 ± 0.01 (Supplementary Table S3). In a rheometer, damping factor (tan *δ*), elastic (*G*′), and viscus (*G*″) moduli are considered as indicators for monitoring the gelation process [29]. On a log scale, typical gel formation kinetics of MCC subjected to UST and DSP for 480 min (8 h) at 25 °C are presented in Figure 3a,b as a gelation profile of *G*′ and *G*″ moduli. *G*′ showed a dramatic increase until it crossed *G*″ at times ranging from 89.00 ± 8.71 to 114.39 ± 3.20 min and from 255.17 ± 2.02 to 280.73 ± 3.69 min of oscillation time at 0 mM and 10 mM of DSP, respectively (Supplementary Table S2). Then *G*′ increased gradually with increasing oscillation time until 480 min. This indicates that formation of the three-dimensional gel network started at those ranges of time and followed with the slow growth of the gel network as the oscillation time progressed [30].

Gelation time (GT) is defined as the time when *G*′ and *G*″ consistently crossover and tan *δ* (the ration of *G*″ to *G*′) = 1 [29]. Figure 3c demonstrates the influence of UST and DSP on the GT of MCC gels. As shown in Figure 3c and Supplementary Table S1, GT decreases significantly with increasing UST (*p* < 0.0001) while it increases with increasing DSP (*p* < 0.0001). There is no interaction effect of UST and DSP. The lowest GT was recorded after 15 min of UST at 0 mM of DSP, where GT reduced from 114.39 ± 3.20 to 83.52 ± 1.61 min (Supplementary Table S2). Consistent with the current results, Nguyen and Anema (2017) found that gelation time of whole milk (total solids of 13% *w*/*w*) decreased markedly from about 85 min to about 45 min after ultrasonication time for 30 min at frequency of 2 and a power of 50 W [41]. During the formation of the acid-induced gel, as the isoelectric point is approached, the net negative charge on casein particles is reduced. Subsequently, the small particles induced by HIUS begin to cross-link to form dimmers. They then grow with time, forming clusters of different sizes. This is followed by the growth of clusters until, at a certain threshold of aggregation, the final clusters are created [29]. It is well known that casein particles initially start to aggregate sooner than the gelation point. The initial aggregation of casein particles manifests itself as an increase in turbidity of solutions over time [29]. The increase in GT with increasing DSP can be attributed to binding colloidal calcium phosphate (CCP) and altering protein–mineral equilibria of casein micelles [15]. This can increase the repulsion between the negatively charged amino acids in the casein micelles, resulting in an increase in hydration and voluminosity of the micelles. Depending on calcium chelator concentration, casein micelles can be dissociated into small clusters, which leads to a decrease in the turbidity of milk dispersions [16] and then an increase in gelation time.

To investigate the combined effect of UST and DSP on the strength of MCC gels, *G*′ values at the last minute of oscillation time (*G*′_480_) were statistically analyzed (Figure 3d). As presented in Figure 3d and Supplementary Table S1, *G*′_480_ is not affected by increasing UST (*p* = 0.62), while it significantly decreases with increasing DSP (*p* < 0.0001). There is an increasing interaction of UST and DSP on *G*′_480_ (*p* = 0.001). By adding 10 mM of DSP, *G*′ _480_ decreased from 108.40 ± 3.29 to 15.78 ± 1.58 Pa (Supplementary Table S2). This indicates that UST did not affect the strength of treated compared to the untreated MCC gels. However, adding DSP resulted in weaker gels. DSP binds micellar calcium ions and changes the mineral balance of the native casein micelles [15,16]. As a result, it reduces cross-linking between casein particles, which results in a gel with a weaker network structure [36]. In contrast with the present results, Nguyen and Anema (2017) investigated the effect of HIUS at a frequency of 22.5 kHz and output power of 50 W for 30 min on the final *G*′ (*G*′ at the end of acidification) of gel formed from whole milk (13% total solids) [41]. They reported that the final *G*′ slightly increased after ultrasonication for 5 and 10 min. By progressing the ultrasound time, *G*′ decreased to about 175 Pa after 30 min. They attributed their results to the denaturation of whey proteins and their interaction with the other components of milk [41].

#### 3.3.2. Frequency Dependence and Viscoelastic Properties

**Figure 4:**
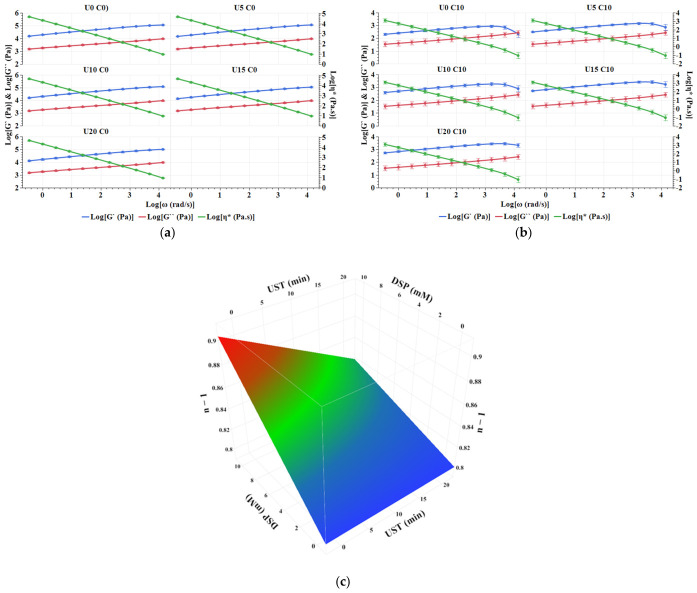
Effect of ultrasound time (UST) and disodium phosphate (DSP) on: (**a**,**b**) oscillation frequency profiles of elastic (*G*′), viscus (*G*″) moduli, and complex viscosity (η*) at 0 and 10 mM of DSP, respectively, (**c**) the slope of log η* (*n* − 1) of micellar casein concentrate: UST (U0 = 0 min, U5 = 5 min, U10 = 10 min, U15 = 15 min, and U20 = 20 min) and DSP concentration (C0 = 0 mM and C10 = 10 mM).

An oscillation frequency test was performed to determine whether UST combined with DSP influences the textural characteristics of MCC gels formed by acid-induced gelation. Figure 4a,b demonstrates the log-log plot of *G*′, *G*″, and η* against angular frequency (*ω*) ranging from 0.63 to 62.83 rad/s (0.1–10 Hz) at 0 and 10 mM of DSP, respectively. As shown in the figure, *G*′, *G*″, and η* are frequency dependent. Both *G*′ and *G*″ increase while η* decreases with increasing frequency. For MCC gels at 10 mM of DSP, *G*′ is greater than *G*″ with increasing *ω* (Figure 4a). This indicates the domination of elastic behaviour of the formed gels, which reflects the typical behaviour for a solid viscoelastic (solid-like) material [30]. Madadlou et al. (2010) reported similar results when they applied ultrasound at a frequency of 24 and 130 Hz and a power of 20.9 W for 60 and 120 min on MCC (3%) [29]. They reported that both *G*′ and *G*″ were frequency dependent and increased with increasing frequency from 0.1 to 10 Hz. They also demonstrated similar shapes and trends to the current results at 0 mM of DSP. The dependency of dynamic moduli and complex viscosity on frequency reflects the relaxation of fewer bonds in the protein network of gels when the timescale of the applied strain is short [29]. However, at 10 mM of DSP, *G*′ decreases after a frequency of 25 rad/s (Figure 4b). This indicates that DSP changed the viscoelastic properties of the formed gels and resulted in less elastic MCC gels compared to MCC gels formed at 0 mM of DSP. Depending on calcium chelator concentration, casein micelles can dissociate into small clusters and release into the aqueous phase. Altering the casein–mineral equilibria has been reported to increase the repulsion between the negatively charged amino acids in the casein micelles and improve micelles’ hydration and voluminosity [15,16]. This leads to an increase in viscus modulus, which results in a weaker network structure and forms a liquid-like gel [11].

To evaluate the effect of UST and DSP on elasticity among MCC gels, the slopes of log η* (*n* − 1) were statistically analyzed (Figure 4c). As shown in Figure 4c and Supplementary Table S1, *n* − 1 significantly decreases with increasing UST (*p* = 0.0007) while it increases with increasing DSP (*p* < 0.0001). There is a decreasing interaction effect of UST and DSP on *n* − 1 (*p* = 0.0033). The lowest value of *n* − 1 was 0.803, which was recorded after 15 min of UST at 0 mM of DSP (Supplementary Table S2). This indicates that UST increased the elasticity of MCC gel networks [30,47]. Independent of DSP, the decrease in *n* − 1 after 15 min of UST is correlated with the GT result (Section 3.3.1), where the lowest GT was also recorded after 15 min of UST, and, as a result, the elasticity of the formed gel increased. In another study conducted by Shen et al. (2017), they reported that the slope of *G*′ versus angular frequency, ranging from 1 to 100 rad/s, was higher in treated whey protein solutions (10% *w*/*v*), indicating that the frequency dependence of *G*′ increased after applying HIUS at a frequency of 20 Hz and amplitude of 40% for 20 min [30]. In the gelation process induced by GDL, two stages are involved: the initial setting up of the gel network, followed by the development of bond strengthening and/or rearrangement [29]. HIUS reduces particle size and increases the surface area of casein micelles, so it exposes the hidden active groups. This facilitates the formation of a dense and uniform gel network [30]. During gel formation, once the casein particles begin to cross-link with each other, the active groups form a continuous network, resulting in a firmer network within gel texture [30].

#### 3.3.3. Gel Firmness

**Figure 5:**
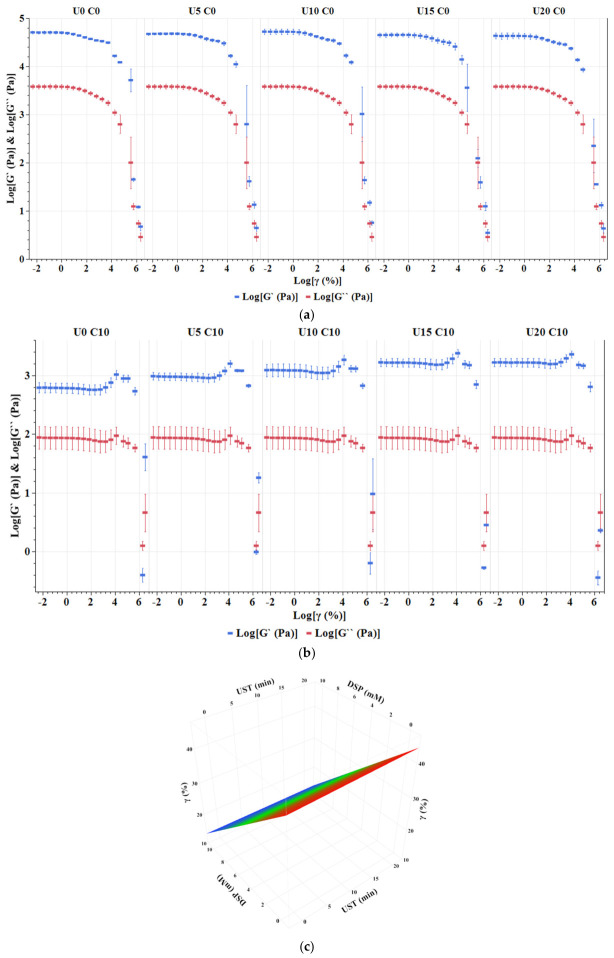
Effect of ultrasound time (UST) and disodium phosphate (DSP) on: (**a**,**b**) oscillation amplitude profiles of elastic (*G*′) and viscus (*G*″) moduli of micellar casein concentrate at 0 and 10 mM, respectively; (**c**) applied strain (*γ*) at the end of linear viscoelastic region (LVR): UST (U0 = 0 min, U5 = 5 min, U10 = 10 min, U15 = 15 min, and U20 = 20 min) and DSP concentration (C0 = 0 mM and C10 = 10 mM).

The oscillation amplitude test is performed to evaluate the linear viscoelastic region (LVR) of the formed gel. LVR is the region where *G*′ values show a non-significant deviation from a constant value. In LVR, the applied strain is not strong enough to irreversibly break the bonds in the gel network. Thus, LVR can be employed as a structural parameter to evaluate the firmness of the formed gel [40]. On a log-log plot, Figure 5a,b illustrates the LVR of the formed MCC gels at 0 and 10 mM, respectively. As shown in the figure, LVRs of MCC gels at 0 mM are higher than those at 10 mM, regardless of UST. LVR ended at γ ranged from 40.36 ± 0.12 to 41.28 ± 0.14% and from 15.82 ± 0.01 to 15.85 ± 0.01% at 0 and 10 mM of DSP, respectively (Supplementary Table S2).

To evaluate the effect of UST and DSP on LVR among MCC gels, *γ* values at the end of LVRs were statistically analyzed (Figure 5c). As presented in Figure 5c and Supplementary Table S1, *γ* significantly increases with increasing UST (*p* < 0.0001) while it decreases with increasing DSP (*p* < 0.0001). There is a decreasing interaction effect of UST and DSP on *γ* (*p* < 0.0001). Independent of DSP, LVR increased by increasing UST, where it extended from 40.36 ± 0.12 to 41.27 ± 0.27% of *γ* after 15 min of UST (Supplementary Table S2). This indicates that UST increased the strength of MCC gels. Consistent with the current results, Nascimento et al. (2023) investigated the effect of HIUS on the gelling properties of mixed protein ratios formed by casein micelles and pea protein at 8% (*w*/*w*) of total protein [40]. HIUS was applied at a constant frequency of 20 Hz and power of 495 W for 15 min at a temperature less than 35 °C. They reported that the pure casein gel presented the largest LVR, which ended at 35% of the applied strain, ranging from 0.1 to 500% [40]. It has been proposed that dissociation of casein micelles into smaller particles due to ultrasonic treatment of casein may influence the structure of the formed gel [1]. Ultrasonic disruption of casein micelles increases their surface area and exposes the active groups hidden in the micelles. During gel formation, once the casein particles begin to cross-link with each other, the active groups form a continuous network. Hydrophobic and electrostatic interactions, as well as hydrogen bonds, are the main forces that stabilize acid gels [29]. The emerged smaller particles could re-aggregate strongly with each other, leading to the formation of a stronger texture of the formed gel network [30]. On the other hand, DSP binds micellar calcium ions and changes the mineral balance of the native casein micelles [15,16]. As a result, it reduces cross-linking between casein particles, which results in a weaker gel [36].

## 4. Conclusions

The combination of ultrasound time (UST) and disodium phosphate (DSP) improved foam properties. Foam capacity increased about 50% after 20 min of UST at 10 mM of DSP. In addition, foam stability was doubled after 10 min of UST at 0 mM. However, emulsifying properties were not enhanced by the combination of UST and DSP. Independent of DSP, increasing UST reduced gelation time and improved the elasticity and firmness of MCC gel networks. High-intensity ultrasound (HIUS) treatment, as a green technology, combined with calcium chelation, might highlight a new approach to improve foaming properties. However, independent of calcium chelation, HIUS treatment is a promising technology to improve the gelling properties of casein micelles. This might extend the application of casein protein in the food industry.

## Figures and Tables

**Table 1:** Effect of ultrasound time (UST) and disodium phosphate (DSP) on the slope (*b*) of model fit foam stability (*FS*), slope (*c*) of model fit emulsion stability index (*ESI*), and the relative change of slope of treated to untreated MCC (*b* or *c*_treated_/*b* or *c*_untreated_): UST (U0 = 0 min, U5 = 5 min, U10 = 10 min, U15 = 15 min, and U20 = 20 min) and DSP concentration (C0 = 0 mM and C10 = 10 mM).

	U0 C0	U5 C0	U10 C0	U15 C0	U20 C0	U0 C10	U5 C10	U10 C10	U15 C10	U20 C10
*b* (1/min) *	0.172 ± 0.012	0.122 ± 0.016	0.075 ± 0.009	0.154 ± 0.012	0.161 ± 0.009	0.160 ± 0.013	0.113 ± 0.008	0.122 ± 0.009	0.128 ± 0.008	0.109 ± 0.005
Relative change of slope (b_treated_/b_untreated_)	1	0.709	0.436	0.895	0.936	0.930	0.657	0.709	0.744	0.634
*c* (1/min) *	0.025 ± 0.003	0.033 ± 0.004	0.043 ± 0.005	0.050 ± 0.007	0.044 ± 0.005	0.036 ± 0.003	0.045 ± 0.005	0.046 ± 0.004	0.047 ± 0.006	0.040 ± 0.006
Relative change of slope (c_treated_/c_untreated_)	1	1.320	1.720	2.000	1.760	1.440	1.800	1.840	1.880	1.600

* Values are presented as *b* or *c* ± SE, where SE is the standard error estimate.

## Data Availability

The original contributions presented in this study are included in the article/Supplementary Materials. Further inquiries can be directed to the corresponding author.

## Supplementary Materials

The following supporting information can be downloaded at https://www.mdpi.com/article/10.3390/foods15122246/s1; Table S1: Effect of UST (ultrasound treatment time) and DSP (disodium phosphate) on FC (foam capacity), EAI (emulsion activity index), GT (gelation time), *G*′_480_ (*G*′ at the last min of oscillation time), n − 1 (slope of log complex viscosity), and γ (applied strain at the end of linear viscoelastic region) from full factorial design; Table S2: Experimental design and value of measured responses: FC (foam capacity), EAI (emulsion activity index), GT (gelation time), *G*′_480_ (*G*′ at the last min of oscillation time), n − 1 (slope of log complex viscosity), and γ (applied strain at the end of linear viscoelastic region) at different UST (ultrasound treatment time) and DSP (disodium phosphate); Table S3: Pre-experimental pH values during acid-induced gelation of micellar casein concentrate (MCC) treated by different ultrasound time (UST) and disodium phosphate (DSP): UST (U0 = 0 min, U5 = 5 min, U10 = 10 min, U15 = 15 min, and U20 = 20 min) and DSP concentration (C0 = 0 mM and C10 = 10 mM).
