# Supplementary material for: Effect of High-Intensity Ultrasound and Calcium Chelation on Functional Properties of Casein Micelles
Source: Foods. 2026 Jun 22;15(12):2246. doi: 10.3390/foods15122246 (PMC13298707; doi:10.3390/foods15122246)
Supplement: Supplementary file 1 [file foods-15-02246-s001.zip › foods-4313701-supplementary.pdf]

Table S1: Effect of UST (ultrasound treatment time) and DSP (disodium phosphate) on FC (foam capacity), EAI (emulsion activity index), GT (gelation time), $G`_{480}$ ($G`$ at the last min of oscillation time), n-1 (slope of log complex viscosity), and γ (applied strain at the end of linear viscoelastic region) from full factorial design

	FC (%)	EAI (m^2/g)	GT (min)	$G`_{480}$ (Pa)	n-1	γ (%)
**Intercept**<br>**p-value**	104<br><0.0001	4.41<br><0.0001	109.82<br><0.0001	105.35<br><0.0001	0.82<br><0.0001	40.66<br><0.0001
**UST**<br>**p-value**	1.93<br>0.0001	-0.12<br>0.0003	-1.31<br><0.0001	0.05<br>0.62	-0.0013<br>0.0007	0.03<br><0.0001
**DSP**<br>**p-value**	1.57<br>0.02	-0.03<br>0.39	16.99<br><0.0001	-8.49<br><0.0001	0.01<br><0.0001	-2.51<br><0.0001
**UST*DSP**<br>**p-value**	0.01<br>0.88	0.003<br>0.54	0.02<br>0.57	0.08<br>0.001	-0.0002<br>0.0033	-0.005<br><0.0001
**Model (p-value)**	0.0003	0.002	<0.0001	<0.0001	<0.0001	<0.0001
**R^2**	0.51	0.42	0.99	0.99	0.87	0.9999
**R^2 adjusted**	0.45	0.36	0.99	0.99	0.86	0.9998
**RMSE**	16.68	0.97	5.33	4.12	0.01	0.14
**Lack of Fit (p-value)**	0.70	0.82	0.01	0.56	0.71	0.49

Note: $p < 0.05$ indicates effect.

Table S2: Experimental design and value of measured responses: FC (foam capacity), EAI (emulsion activity index), GT (gelation time), $G`_{480}$ ($G`$ at the last min of oscillation time), n-1 (slope of log complex viscosity), and γ (applied strain at the end of linear viscoelastic region) at different UST (ultrasound treatment time) and DSP (disodium phosphate)

UST (min)	DSP (mM)	FC (%)	EAI (m^2/g)	GT (min)	$G`_{480}$ (Pa)	n-1	γ (%)
0	0	110.00 ± 13.23	4.48 ± 0.34	114.39 ± 3.20	108.40 ± 3.29	0.809 ± 0.004	40.36 ± 0.12
5	0	110.00 ± 20.00	4.09 ± 1.46	102.39 ± 2.49	106.04 ± 2.91	0.804 ± 0.001	40.67 ± 0.28
10	0	121.67 ± 14.43	3.60 ± 1.37	94.10 ± 4.23	110.42 ± 7.24	0.807 ± 0.005	40.99 ± 0.09
15	0	126.67 ± 28.43	2.18 ± 0.62	83.52 ± 1.61	103.39 ± 5.56	0.803 ± 0.002	41.27 ± 0.27
20	0	148.33 ± 20.21	2.39 ± 1.10	89.00 ± 8.71	101.16 ± 7.33	0.804 ± 0.006	41.28 ± 0.14
0	10	115.00 ± 13.23	4.41 ± 1.64	280.73 ± 3.69	15.78 ± 1.58	0.894 ± 0.019	15.82 ± 0.01
5	10	133.33 ± 7.64	2.98 ± 1.08	269.14 ± 2.92	19.16 ± 1.48	0.865 ± 0.022	15.81 ± 0.01
10	10	146.67 ± 11.55	2.83 ± 0.35	269.31± 3.00	21.41 ± 2.74	0.872 ± 0.015	15.84 ± 0.02
15	10	136.67 ± 23.09	2.67 ± 1.01	258.51 ± 5.37	24.36 ± 2.08	0.852 ± 0.023	15.84 ± 0.01
20	10	163.33 ± 11.55	2.31 ± 0.09	255.17 ± 2.02	24.41 ± 2.05	0.841 ± 0.013	15.85 ± 0.01

Note: Data presented as mean ± standard deviation

Table S3: Pre-experimental pH values during acid-induced gelation of micellar casein concentrate (MCC) treated by different ultrasound time (UST) and disodium phosphate (DSP): UST (U0 = 0 min, U5 = 5 min, U10 = 10 min, U15 = 15 min, and U20 = 20 min) and DSP concentration (C0 = 0 mM and C10 = 10 mM).

**Treatment**<br>**Time (min)**	**U0 C0**	**U5 C0**	**U10 C0**	**U15 C0**	**U20 C0**	**U0 C10**	**U5 C10**	**U10 C10**	**U15 C10**	**U20 C10**
**30**	5.55±0.01	5.55±0.00	5.54±0.01	5.54±0.02	5.53±0.01	5.63±0.01	5.63±0.00	5.63±0.01	5.63±0.02	5.61±0.02
**60**	5.43±0.02	5.43±0.00	5.42±0.00	5.42±0.03	5.41±0.02	5.50±0.01	5.50±0.02	5.50±0.00	5.50±0.00	5.48±0.03
**90**	5.33±0.08	5.32±0.08	5.32±0.09	5.31±0.07	5.30±0.08	5.38±0.11	5.38±0.10	5.37±0.10	5.37±0.11	5.32±0.02
**120**	5.23±0.03	5.22±0.04	5.21±0.03	5.22±0.02	5.19±0.01	5.29±0.06	5.31±0.05	5.30±0.05	5.29±0.05	5.28±0.03
**150**	5.15±0.02	5.15±0.01	5.14±0.01	5.14±0.02	5.12±0.02	5.24±0.01	5.23±0.00	5.22±0.01	5.23±0.03	5.21±0.01
**180**	5.10±0.01	5.09±0.01	5.08±0.00	5.09±0.02	5.06±0.01	5.19±0.02	5.19±0.01	5.18±0.00	5.18±0.03	5.17±0.02
**210**	5.04±0.01	5.03±0.00	5.03±0.01	5.03±0.02	5.01±0.02	5.15±0.04	5.14±0.00	5.13±0.01	5.14±0.04	5.13±0.02
**240**	*4.98±0.01	*4.97±0.01	*4.97±0.01	*4.97±0.01	*4.93±0.01	5.21±0.13	5.08±0.02	5.06±0.04	5.08±0.06	5.06±0.01
**270**	4.94±0.00	4.94±0.01	4.95±0.04	4.94±0.04	4.90±0.01	5.04±0.04	5.03±0.02	5.03±0.02	5.04±0.04	5.04±0.02
**300**	4.93±0.02	4.90±0.01	4.91±0.01	4.90±0.04	4.86±0.02	5.00±0.03	5.01±0.01	5.00±0.01	5.01±0.04	4.99±0.01
**330**	4.91±0.04	4.84±0.01	4.88±0.03	4.87±0.05	4.82±0.03	4.98±0.03	4.99±0.02	4.96±0.04	4.99±0.03	4.97±0.02
**360**	4.90±0.07	4.82±0.01	4.85±0.02	4.80±0.00	4.79±0.01	*4.87±0.10	*4.96±0.01	*4.93±0.04	*4.94±0.01	*4.95±0.04
**390**	4.87±0.06	4.77±0.00	4.82±0.02	4.81±0.04	4.77±0.02	4.94±0.04	4.95±0.04	4.91±0.04	4.96±0.04	4.92±0.02
**420**	4.84±0.06	4.75±0.02	4.80±0.04	4.79±0.06	4.75±0.03	4.95±0.01	4.92±0.03	4.9±0.03	4.96±0.03	4.89±0.01
**450**	4.81±0.06	4.76±0.01	4.80±0.01	4.76±0.04	4.74±0.04	4.99±0.05	4.92±0.04	4.91±0.01	4.96±0.03	4.88±0.02
**480**	4.83±0.09	4.76±0.04	4.81±0.04	4.78±0.10	4.73±0.04	4.94±0.08	4.90±0.06	4.92±0.00	4.99±0.04	4.91±0.04

*Gel formation
